# Operant control and call usage learning in African elephants

**DOI:** 10.1098/rstb.2020.0254

**Published:** 2021-10-25

**Authors:** Angela S. Stoeger, Anton Baotic

**Affiliations:** Mammal Communication Laboratory, Department of Behavioral and Cognitive Biology, University of Vienna, Vienna, Austria

**Keywords:** African elephants, vocal usage learning, vocal learning, vocal communication

## Abstract

Elephants exhibit remarkable vocal plasticity, and case studies reveal that individuals of African savannah (*Loxodonta africana*) and Asian (*Elephas maximus*) elephants are capable of vocal production learning. Surprisingly, however, little is known about contextual learning (usage and comprehension learning) in elephant communication. Usage learning can be demonstrated by training animals to vocalize in an arbitrary (cue-triggered) context. Here we show that adult African savannah elephants (*n* = 13) can vocalize in response to verbal cues, reliably producing social call types such as the low-frequency rumble, trumpets and snorts as well as atypical sounds using various mechanisms, thus displaying compound vocal control. We further show that rumbles emitted upon trainer cues differ significantly in structure from rumbles triggered by social contexts of the same individuals (*n* = 6). Every form of social learning increases the complexity of a communication system. In elephants, we only poorly understand their vocal learning abilities and the underlying cognitive mechanisms. Among other research, this calls for controlled learning experiments in which the prerequisite is operant/volitional control of vocalizations.

This article is part of the theme issue ‘Vocal learning in animals and humans’.

## 1. Introduction

Along with whales, dolphins, seals and bats, elephants belong to a diverse and dissimilar group of non-human mammals proven capable of vocal production learning, i.e. of structurally modifying signals as a result of auditory experience [1,2]. The vocal system of elephants is characterized by its plasticity, exhibiting a grading between call types, call-type combinations and context-dependent within-call type flexibility [3]. African elephants use vocalizations with fundamental frequencies (F0) in the infrasonic range (rumbles) for short- and long-distance communication. When aroused, they produce higher pitched trumpets, snorts, and roars [3].

We have only scratched the surface of vocal production mechanisms in elephants, but it has become increasingly clear that their acoustic flexibility reflects special nasopharyngeal morphological structures. The elephant trunk plays a crucial role in sound production [4,5]. While roars seem to be laryngeal as well, trumpets and snorts seem to be produced by blasts out of the trunk.

Contextual learning, another form of social learning in animal communication, has not, to our knowledge, been addressed in elephants so far. Contextual learning affects the behavioural context of a pre-existing signal and is further distinguished into comprehension and usage learning [1]. Comprehension learning occurs when an individual extracts a novel meaning from a signal based on experience. Usage learning, by contrast, occurs when an individual learns to produce an existing signal in a novel context [1], which might be relevant for young animals learning how and when to use vocalizations, or in other age-related periods that require linking a new context with an existing vocalization. In non-human mammals, usage learning has been verified for example in whales and dolphins [6], pinnipeds [7,8], monkeys and apes [9], or bats [10]. Usage learning can be best demonstrated if an animal is able to vocalize in response to a conditioning stimulus [1,7]. Here, we show that African elephants are capable of producing sounds in response to different discriminative verbal cues.

## 2. Material and methods

The elephants (*Loxodonta africana*, *n* = 13) were observed in five facilities in Botswana, South Africa, Germany and Austria (table 1) from June 2014 until June 2020, and again in February and March 2021. In the European facilities, the elephants are managed in a protected contact system (in which the handlers and the elephants are separated by a barrier), in South Africa and Botswana the handlers have direct contact with the elephants. Training methods differed considerably between facilities (for details see the electronic supplementary material), and none of the elephants was trained specifically for this study. In all facilities, the training is based on positive reinforcement with food rewards as the primary reinforcer. At the European zoos, elephants are exposed to a standardized target and clicker (secondary reinforcer) training. Following the verbal cue, the elephants are supposed to vocalize once. Thereafter, a second, different cue, elicited a second type of vocalization, etc. In Botswana and South Africa, instead of a clicker, verbal praise (e.g. ‘good boy') and patting is used as secondary reinforcer.

**Table 1.** Call types, their supposed production mechanism, the vocal cues and the identity (ID) of the elephants producing the particular sounds. (Distinction made between sounds naturally occurring within the vocal repertoire, alterations from a natural vocalization type and atypical sounds.)

call type	natural/atypical	vocal cue^a^	ID elephants, male/*female*	sound production mechanism
Botswana: living with elephants foundation				
rumble	natural	‘talk'	Jabu (electronic supplementary material, video S1), *Morula*	passive vocal fold vibration, emitted nasally
trumpet	natural	‘trumpet’	Jabu (electronic supplementary material, video S2)	trunk blast
soft snort	natural	‘how would you do?’	Jabu	trunk exhalation
rasberry sound (like a soft rumble)	alteration from natural rumble	‘rasberry’	Jabu, *Morula*^b^	emitted nasally
snort	natural	‘blast’	Jabu, *Morula*	trunk blast (similar to the trumpet, but with less power and force)
throb sound	atypical	‘gluck gluck’	Jabu (electronic supplementary material, video S3)	contraction of muscles at the forehead
high-frequency sound	atypical	‘squeak’	Jabu (electronic supplementary material, video S4), *Morula*	nasal tissue vibration during inhalation
South Africa: adventures with elephants				
rumble	natural	‘talk’	Chova, Chishuru	passive vocal fold vibration, emitted nasally
South Africa: elephant whispers				
rumble	natural	‘talk’	Tembo, Medwa, Ziziphus, Shamwari	passive vocal fold vibration, emitted nasally
Germany: Dresden Zoo				
trumpet^c^	natural	‘trumpet’	*Mogli* (electronic supplementary material, video S5)*, Drumbo* (electronic supplementary material, video S6)	trunk blast
snort	natural	‘snore’	*Mogli* (electronic supplementary material, video S5)	trunk blast
high-frequency sound	atypical	‘squeal’	*Sawu* (electronic supplementary material, video S7)	nasal tissue vibration during inhalation
oral burst	atypical	‘speak’	*Drumbo* (electronic supplementary material, video S6), *Mogli* (electronic supplementary material, video S5), *Sawu* (electronic supplementary material, video S7)	air blocked by a posterior obstruction of oral chamber, then released suddenly, causing a burst of sound
Austria: Vienna Zoo				
trumpet	natural	‘Laut’	*Iqhwa* (electronic supplementary material, video S8)	trunk blast

^a^Often the elephant's name is added before the cue, e.g. ‘Chova talk’, in all facilities, in the South African institutions the word ‘louder’, in Dresden, the word ‘feste’ (=strong) is sometimes added to emphasize the cue, e.g. ‘Mogli feste trumpet’.

^b^Morula's sounds in response to the rasberry cue resemble in structure the throb sounds produced by Jabu.

^c^To be treated with caution, missing data on response accuracy.

Recordings were conducted using a Neumann KM183 microphone connected to a Sound Devices 722 or 633 (frequency response of both systems: 10 Hz–40 kHz) at 48 kHz sampling rate and 16-bit, and a Sony FD53 camcorder.

All cue vocalizations were recorded during routine training sessions. During that data collection, we also recorded rumbles of six male elephants during social interactions at the South African facilities (see the electronic supplementary material for details) and used those to compare the acoustic structure of rumbles on cue with social rumbles of the same individuals.

### (a) Data analysis

Acoustic data were annotated using a customized annotation tool from S_Tools STx [11]. Each call type was identified based on overall acoustic structure and sound quality. The start and end of each vocalization were tagged and the corresponding annotations were added.

A detailed acoustic and statistical analysis was conducted for social rumbles and rumbles on cue of six male individuals. Here, the F0 parameter was analysed using a customized semi-automatic analysis tool in Matlab [12] and formant 1 using S_Tools STx. Our dataset comprised a total of 208 rumbles (with 107 cue and 101 social rumbles, balanced by individuals and context) from six African elephant bulls. To test whether the acoustic structure of social rumbles differs from rumbles on cue, we performed a permuted discriminant function analysis (pDFA) [13] (see the electronic supplementary material for details).

## 3. Results

**Figure 1.**
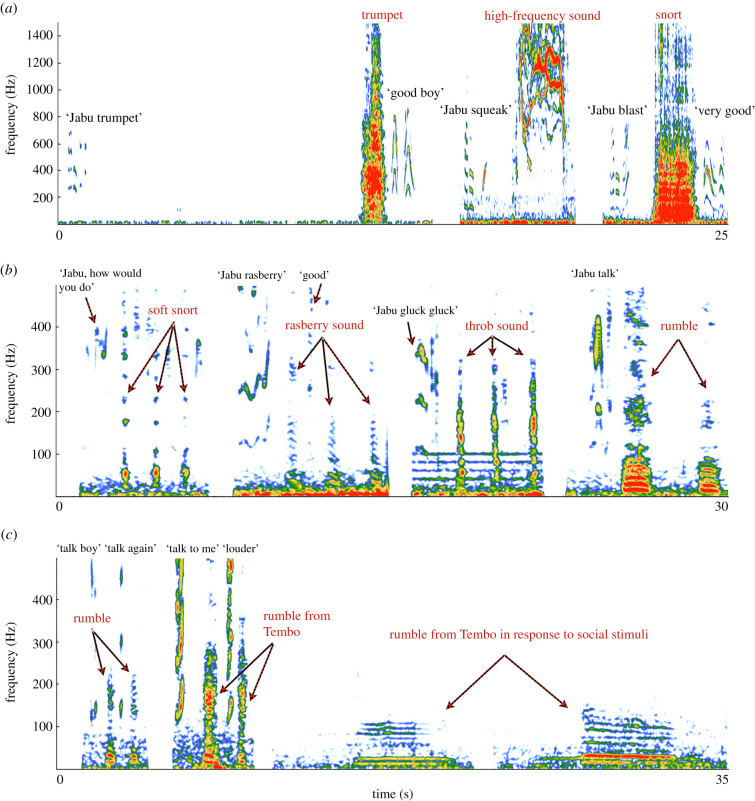
Graphical representation of sounds, the corresponding verbal cues and reinforcements by human carer. (*a*,*b*) show the seven sounds produced by Jabu, graph (*c*) displays rumbles on cue (by Ziziphus and Tembo) compared with rumbles by Tembo produced in response to social stimuli (see the electronic supplementary material, sound file SF1–SF3). It takes 17.5 s before Jabu starts to trumpet following the ‘Jabu trumpet' cue (*a*).

We documented 13 African elephants that vocalized in response to verbal cues, and reliably produced rumbles, trumpets, snorts and alterations from those. We also found that some individuals produced novel, high-frequency sounds that are not part of the natural African elephant repertoire (table 1). Since we did not know to what extent unusual sounds were initially rewarded during training, these are either a result of selective shaping during training or of an invention process that elephants used to fulfil the training requirements. The acoustic structure of the vocalization types and the corresponding verbal cues are exemplified in spectrograms (figure 1*a*–*c*), and videos of training sessions are given (see the electronic supplementary material, videos S1–S8). Table 2 reports information on the number of trials and the success rate for each individual and each call type.

**Table 2.** Information on the number of trails (i.e. number of vocal cues given), the numbers of correct responses and the success rate in % for each individual and each call type (HFS, high-frequency sound). In addition, the numbers of correct responses to the release cues (and success rates) are given for Jabu and Morula.

individual	call type	*n vocal cues* (*n* sessions)	*n* correct responses	success rate	*n* correct responses to *release cue* (success rate)
Sawu	HFS	30 (in 8 sessions)	25	83.3%	
Sawu	oral burst	31 (in 8 sessions)	25	80.6%	
Drumbo	oral burst	31 (in 8 sessions)	30	96.7%	
Mogli	snort	30 (in 8 sessions)	28	93.3%	
Mogli	oral burst	32 (in 8 sessions)	27	84.4%	
Iqhwa	trumpet	24 (in 15 sessions)	20	83.3%	
Jabu^a^	rumble	6 (in 5 sessions)	6	100%	6 (100%)
Jabu^a^	trumpet	7 (in 5 sessions)	6	85.7%	6 (100%)
Jabu^a^	soft snort	7 (in 5 sessions)	7	100%	6 (100%)
Jabu^a^	soft rumble	11 (in 5 sessions)	11	100%	11 (100%)
Jabu^a^	throb	8 (in 5 sessions)	8	100%	8 (100%)
Jabu^a^	snort	6 (in 5 sessions)	6	100%	6 (100%)
Jabu^a^	HFS	10 (in 5 sessions)	10	100%	10 (100%)
Morula^a^	rumble	8 (in 5 sessions)	8	100%	8 (100%)
Morula^a^	HFS	28 (in 5 sessions)	22	78.6%	22 (100%)
Morula^a^	soft snort	9 (in 5 sessions)	9	100%	9 (100%)
Chishuru	rumble	15 (in 5 sessions)	13	86.7%	
Chova	rumble	20 (in 5 sessions)	20	100%	
Tembo	rumble	19 (in 5 sessions)	19	100%	
Medwa	rumble	18 (in 5 sessions)	16	88.7%	
Shamwari	rumble	20 (in 5 sessions)	19	100%	
Ziziphus	rumble	20 (in 5 sessions)	20	100%	

^a^Following the cue, the elephants are trained to remain vocalizing until released with the release cue (‘alright’).

Jabu, an adult male, produced seven different vocalizations on cue (figure 1*a*,*b*); his accompanying female, Morula, produced four types of calls. When Jabu and Morula receive the specific cue, they start producing that vocalization in a repetitive manner until the trainer says ‘alright', at which point the elephants stop vocalizing reliably (table 2). Overall, Jabu responded correctly and immediately on cue in 96.4% of the cases, with the only inaccuracy once following a trumpet cue, that had to be repeated. Morula responded correctly in 86.7% and as Jabu, never confused cues and vocalization types, but sometimes responded only after the cue was repeated (which is counted as an inaccuracy). Two females at Dresden Zoo were further found to be capable of producing two different cue-stimulated call types (tables 1 and 2) each. Sawu produced high-frequency sounds with an accuracy of 83.3% and oral bursts with an 80.6% success rate. Mogli correctly emitted snorts in 93.3%, and oral bursts in 84.4%. Drumbo produced only oral bursts on cue with an accuracy of 96.8%. Mogli and Drumbo have further been observed to trumpet on cue, but data on response accuracy are not available. Iqhwa from the Vienna Zoo produced trumpets on cue and responded correctly in 83.3%. We further provide tables for response accuracy per training session for each individual and each call cue (also providing information on the types of mistakes) in the electronic supplementary material, tables S1–S6.

The elephants at the South African institutions were trained to rumble on cue (with ‘talk' being the main cue, but variations were observed, e.g. ‘talk boy', ‘talk louder’ or ‘talk to me', figure 1*c*). Overall, the elephants vocalized on cue correctly in 95.6% (see table 2 for individual success rates). Naturally, rumbles are used by these males in social contexts and have been shown to encode information on individuality, maturity and sex [14,15]. Call duration ± s.d. was considerably shorter in cue rumbles (1.124 ± 0.802 s; *n* = 107) versus social rumbles (3.733 ± 1.787 s; *n* = 101). The pDFA resulted in 87.5% correct classification (*p* = 0.013) and 91.1% correct cross-validated classification (*p* = 0.013), revealing a significant difference in acoustic structure between rumbles emitted socially or on cue (figure 1*c*). For details on response accuracy, contexts of social rumbles, acoustic measurements and statistics see the electronic supplementary material, tables S7–S11.

## 4. Discussion

For an experimental demonstration of call usage learning, Shapiro *et al*. [7] define that, first, an animal has to reliably produce a call in response to a specific cue. Even more convincing evidence is that an animal remains silent or stops vocalizing on a different cue. Jabu and Morula learned to stop maintaining vocalizing in response to the specific ‘alright' cue. The most complex level of usage learning involves an animal emitting different call types in response to distinct cues [7]. In our dataset, four elephants produce two or more different call types on cue. Jabu, emitting seven call types performs highly accurately (96.4% correct), and the success rates of over 80% in the other individuals suggest that African elephants exhibit a complex level of usage learning. Variable training techniques were used in the different facilities, and none of the elephants was trained for the purpose of this study. This might have negatively influenced the level of accuracy for vocalizing on cue. While Jabu started to learn as a calf, the elephants from Dresden Zoo as well as Morula learned the sounds on cue as adults. Future controlled experiments with objective trainer guidance might yield more information on how training methods and other aspects such as e.g. the elephant's age, gender or personality affect learning speed and accuracy of call production.

The trumpet seems particularly difficult for elephants to produce on cue. For an elephant to trumpet naturally, context needs to be linked to a specific internal state. Elephants trumpet in situations such as bonding ceremonies (electronic supplementary material, video S9) or play behaviour if in a state of high arousal (i.e. a trumpet is not always associated with greeting or play) [3]. Accordingly, when trumpeting on cue, the elephant probably must coordinate brain regions associated with the arousal regulation of vocal production and the pathway involved in volitional vocal control. This potential cognitive effort might be reflected by the observation that the elephants need a considerable time (up to 17 s in one training session by Jabu (figure 1*a*)) to execute the trumpet (see the electronic supplementary material, video S2 (Jabu) and S5 (Mogli)).

Vocalizations on cue never elicited a behavioural response by group members close by. In the case of the rumble, we had sufficient data on social rumbles and rumbles on cue of the same individuals to determine that the acoustic structure of the former differs significantly from the latter. This might be a training artefact because training was not specifically focused and the animals might have been unintentionally reinforced for certain call features, such as short duration. On the other hand, the social rumbles encode a lot of information in the time and frequency domain [3,14,15], which hints at the influence of call motivation and context on elephant vocalizations, apart from their vocal control ability.

The next step is to deepen our understanding of contextual and vocal production learning and the underlying cognitive mechanisms (including *Elephas maximus*), as many open questions remain. Each form of social learning increases the complexity of a communication system. Elephants naturally use their vocal skills for individual recognition [16], group cohesion and coordination with the rumble being the dominating call type [3]. Neonates rumble (though different from adults) soon after birth [3,17]. Therefore, this call type does not *per se* seem to require vocal production learning in order to be produced (maybe it is necessary later on when developing individual or family specific call features), but elephant calves and juveniles might have to learn how and when to use specific rumble variants in order to negotiate within the complex social network of elephant society. The combined ability of vocal production and usage learning highlights the value of elephants—–highly social, long-lived and terrestrial mammals—as a study species for specifically addressing the behavioural and ecological relevance of vocal learning.

## Data accessibility

Refer to the electronic supplementary material [19] for videos S1–S7, sound files SF1–SF3 and text and tables S1–S3 for more information.
